# Life- threatening hemothorax due to the inferior pulmonary ligament injury without obvious organ injuries: a case report

**DOI:** 10.1186/s13019-015-0243-8

**Published:** 2015-03-21

**Authors:** Jae Jun Kim, Yong Hwan Kim, Si Young Choi, Seong Cheol Jeong, Seok Whan Moon

**Affiliations:** 1 Department of Thoracic and Cardiovascular Surgery, Uijeongbu St. Mary’s Hospital, The Catholic University of Korea College of Medicine, 480-717 Geumo-dong, Uijeongbu, Gyeonggi-do South Korea; 2 Department of Thoracic and Cardiovascular Surgery, St. Paul Hospital, The Catholic University of Korea College of Medicine, Seoul, South Korea

**Keywords:** Trauma, Hemothorax, Inferior pulmonary ligament

## Abstract

Traumatic hemothorax is usually associated with obvious organ injuries, such as rib fractures, pulmonary injuries, and other mediastinal injuries. We present a rare case in which a 42-year- old Korean man who fell off of a roof, approximately 3 meters in height, resulting in a life-threatening hemothorax without obvious injuries to the thoracic organs. Chest CT showed a large amount of hemothorax in the right side of the thoracic cavity, and an active bleeding, presumably from the posterior intercostal or the phrenic artery, with a focal aneurysmal change. The emergency thoracotomy was performed to bring the active bleeding under control. The operative findings showed there were only the inferior pulmonary ligament tears, and the active bleeding from it. The postoperative course was uneventful and the patient was discharged without any complications. We should consider the inferior pulmonary ligamental injury as one of causes for traumatic hemothorax.

## Background

Traumatic hemothorax is usually associated with obvious organs injuries, such as such as rib fractures, pulmonary injuries, and other mediastinal injuries [1,2]. We present a rare case, in which an emergent thoracotomy was performed for the life-threatening hemothorax due to the inferior pulmonary ligament injury without obvious organs injuries.

## Case presentation

**Figure 1:**
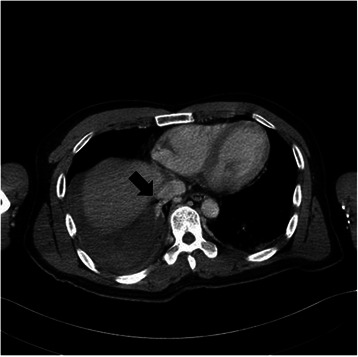
**Chest CT shows that a large amount of hemothorax and an active bleeding, presumably from the posterior intercostal or the phrenic artery, with focal aneurysmal changes.** In addition, no injuries to the thoracic organs, including ribs, the lung, the mediastinum, or the diaphragms, are observed.

**Figure 2:**
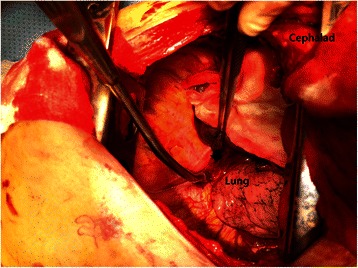
**Operative findings of the thoracotomy show only the inferior pulmonary ligament tears, and active bleeding from the tears.** There are no obvious organ injuries including ribs, the lung, mediastinal structures. (Arrow: inferior pulmonary ligament tears and ruptured artery).

A 42-year-old Korean man visited our hospital emergency department for a heavy trauma to the right chest. He fell off of a roof approximately 3 meters in height during his work. The primary survey revealed he was conscious, alert, and oriented. Vital sign was tachypneic (25/min), tachycardic (110/min) and normotensive (110/70 mm Hg). Chest CT showed a large amount of the right hemothorax and the active bleeding presumably from the posterior intercostal or the phrenic artery with a focal aneurysmal change (Figure 1). However, no obvious organ injuries, including rib fractures, lung injuries, diaphragm ruptures, or other mediastinal structural injuries, were not found. Due to rapid deterioration, the emergency thoracotomy was performed to bring the active bleeding under control. The operative findings showed that there were more than 1000 cc hematoma around the inferior pulmonary ligament, and there were no obvious organ injuries, including lung injuries, diaphragmatic injuries, rib fractures, or other mediastinal structural injuries. Only the inferior pulmonary ligament tears, and active bleeding from the tearing, were observed (Figure 2). After removal of the hematoma, the inferior ligament tearing was repaired with 3-0 black silk stitches. The postoperative course was uneventful, and the patient was discharged without any complications. He is being followed up without any complication 1 year after the surgery.

### Discussion

Most common cause of a hemothorax is trauma [1-3]. Traumatic hemothorax is usually consequence of other organ damage, such as chest wall, the lung paremchyme and the tracheobronchial tree, the esophagus, the diaphragm, and the heart or great vessels [1,2]. Thoracic distortion and shearing after blunt trauma to the chest can result in the injuries in any one or all structures of the thoracic cavity and the chest wall [3]. Also, laceration of internal vessels by blunt trauma can occasionally result in hemothorax [1-3].

This present case, in which a massive, active, life-threatening hemothorax developed, in spite of the absence of injuries of other components of the chest wall, and thoracic structures, is rare. Also, it required an emergency thoracotomy in order to control the unstable condition due to the active bleeding. At first, we considered the use of VATS. But we chose the open thoracotomy rather than VATS because the patient was in rapid deterioration due to massive and active bleeding. To the best of our knowledge, the present report is the first ever report about a hemothorax due to inferior pulmonary ligament tears without obvious organ injuries after blunt trauma. At first, the diaphragmatic injury, or the phrenic artery laceration, were suspected for the hemothorax, but the operative findings showed no the other component injuries in the chest wall and the thoracic cavity. Only the inferior pulmonary ligament tears, and the active bleeding from the tears, were observed. This injury was thought to be the result from a remarkable pressure gradient application to the inferior pulmonary ligament.

The inferior pulmonary ligament comprises of a double membrane of pleura that drapes caudally from the lung root and loosely tethers the medial side of the lower lobe of the lung to the mediastinum [4]. The inferior pulmonary ligament can be torn by a remarkable pressure gradient application, such as traction and compression, because it anchors the lower lobe to the mediastinum and to the diaphragm. The space between the two membranes of the inferior pulmonary ligament contains loose connective tissue, small arterial branches from bronchus and esophagus, some venules draining into the superior diaphragmatic veins, and some lymphatic vessels and nodes emptying into the lower lobes of the lung and lower third of the thoracic esophagus [4]. Therefore, a massive, active, and life- threatening hemothorax can be developed from inferior pulmonary ligament injuries. In addition, the possibility of the malformation of the vasculature in the inferior pulmonary ligament should be considered.

Contrast-enhanced CT is generally considered to be mandatory in order to identify and localize the bleeding foci by detection of extravasations of a contrast agent, or aneurysmal changes of the bleeding foci [2,3,5]. In the present case, chest CT showed the active bleeding from the laceration of artery in the inferior pulmonary ligament.

## Conclusion

We report a rare case of a life- threatening hemothorax due to the inferior pulmonary ligament tearing without obvious organ injuries after blunt trauma. The inferior pulmonary ligament injury without obvious organ injuries should be considered to be one of causes for traumatic hemothorax.

## Consent

Written informed consent was obtained from the patient for publication of this Case report and any accompanying images. A copy of the written consent is available for review by the Editor-in-Chief of this journal. This case study was approved by Institutional Review Board for Uijeongbu St. Mary’s Hospital (UC14ZISE0079).
